# Correlation Between Increased Homing Flight Duration and Altered Gene Expression in the Brain of Honey Bee Foragers After Acute Oral Exposure to Thiacloprid and Thiamethoxam

**DOI:** 10.3389/finsc.2021.765570

**Published:** 2021-12-10

**Authors:** Verena Christen, Daniela Grossar, Jean-Daniel Charrière, Michael Eyer, Lukas Jeker

**Affiliations:** ^1^University of Applied Sciences and Arts Northwestern Switzerland, School of Life Sciences, Muttenz, Switzerland; ^2^Agroscope, Swiss Bee Research Center, Bern, Switzerland; ^3^Laboratory of Soil Biodiversity, University of Neuchâtel, Neuchâtel, Switzerland

**Keywords:** neonicotinoids, homing flight activity, foragers, gene expression pattern, gene expression analysis

## Abstract

Neonicotinoids as thiamethoxam and thiacloprid are suspected to be implicated in the decline of honey bee populations. As nicotinic acetylcholine receptor agonists, they disturb acetylcholine receptor signaling in insects, leading to neurotoxicity and are therefore globally used as insecticides. Several behavioral studies have shown links between neonicotinoid exposure of bees and adverse effects on foraging activity, homing flight performance and reproduction, but the molecular aspects underlying these effects are not well-understood. In the last years, several studies through us and others showed the effects of exposure to neonicotinoids on gene expression in the brain of honey bees. Transcripts of acetylcholine receptors, hormonal regulation, stress markers, detoxification enzymes, immune system related genes and transcripts of the energy metabolism were altered after neonicotinoid exposure. To elucidate the link between homing flight performance and shifts in gene expression in the brain of honey bees after neonicotinoid exposure, we combined homing flight activity experiments applying RFID technology and gene expression analysis. We analyzed the expression of endocrine factors, stress genes, detoxification enzymes and genes linked to energy metabolism in forager bees after homing flight experiments. Three different experiments (experiment I: pilot study; experiment II: “worst-case” study and experiment III: laboratory study) were performed. In a pilot study, we wanted to investigate if we could see differences in gene expression between controls and exposed bees (experiment I). This first study was followed by a so-called “worst-case” study (experiment II), where we investigated mainly differences in the expression of transcripts linked to energy metabolism between fast and slow returning foragers. We found a correlation between homing flight duration and the expression of *cytochrome c oxidase subunit 5A*, one transcript linked to oxidative phosphorylation. In the third experiment (experiment III), foragers were exposed in the laboratory to 1 ng/bee thiamethoxam and 8 ng/bee thiacloprid followed by gene expression analysis without a subsequent flight experiment. We could partially confirm the induction of *cytochrome c oxidase subunit 5A*, which we detected in experiment II. In addition, we analyzed the effect of the feeding mode (group feeding vs. single bee feeding) on data scattering and demonstrated that single bee feeding is superior to group feeding as it significantly reduces variability in gene expression. Based on the data, we thus hypothesize that the disruption of energy metabolism may be one reason for a prolongation of homing flight duration in neonicotinoid treated bees.

**Graphical Abstract:**
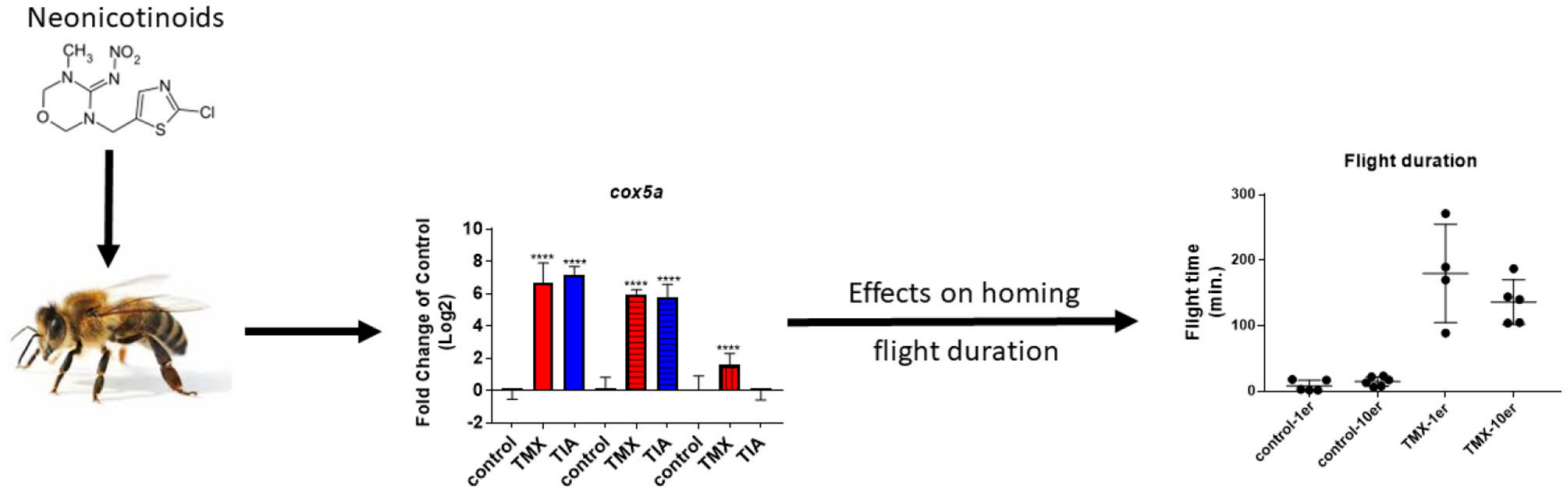
Possible correlation between gene expression and homing flight duration of honey bee foragers exposed to neonicotinoids.

## Introduction

Over the last 50 years, the number of species of wild bees and other pollinating insects has declined [reviewed in: (1)]. For some species, the number of individuals has extremely decreased, other species have become extinct [reviewed in: (1)]. Furthermore, the number of managed honey bee colonies has declined in North America and in many European countries (2). In China and Argentina, on the other hand, the number of managed honey bee colonies has increased (3–5). There are alarming reports of high colony losses in managed honey bees (colony collapse disorder CCD) from several areas of the world (6). Several factors including CCD are suspected to cause the decline of bees. One of them is habitat loss and the resulting lack of plants as food source. Furthermore, diseases caused by bacterial and viral infections, and parasites e.g., the parasitic mite *Varroa destructor* contribute to the decline of bees [reviewed in: (1)]. Also the exposure to pesticides was suggested as driver of the bee decline worldwide (7–9).

The large-scale use of herbicides resulting in fewer attractive flowers being available and insecticides used to protect crops from pests, can have direct adverse and toxic effects on beneficial insects and pollinators such as bees [reviewed in: (10); reviewed in: (1)]. Bees can be exposed to pesticides directly during spray application (droplets, dust, and drift) and *via* contaminated nectar, honey and pollen (11). In most cases, pollen and nectar are contaminated not only with one, but also with a cocktail of different pesticides (12, 13). Sanchez-Bayo and Goka (13) identified in their metastudy on pesticide residue surveys performed on pollen, honey and wax samples from European and US honey bee colonies a total of 161 different pesticides. Of these, insecticidal neonicotinoids (such as clothianidin, imidacloprid, and thiamethoxam) and organophosphates (like phosmet and chlorpyrifos) pose severe risks to bees (13, 14).

Neonicotinoids in particular are associated with the decline of bees. They are neurotoxic compounds and bind as agonists to the nicotinic acetylcholine receptor and cause hyperstimulation, paralysis and death (15). Besides the acute toxicity of neonicotinoids, their sublethal effects might also play a role in the decline of bees [reviewed in: (16)]. Sublethal effects by neonicotinoids include adverse effects on the immune system (17), impaired orientation and memory formation (18), reduced colony size and negative effects on larval development (19, 20), altered homing flight activity (21, 22), negative effects on self-grooming (23) and changes in gene expression (24–28). Negative effects of neonicotinoids in terms of prolongation of flight times and flight activities of foragers are known (22, 29). The radiofrequency identification (RFID) technology is a common method to analyze negative effects of neonicotinoids on homing flight performance of honey bees (30–34). By applying RFID technology, it was possible to show that exposure of foragers to thiamethoxam resulted in a reduced return rate and in a prolonged homing flight duration of returning bees (35).

To study sublethal effects of pesticides on gene expression in honey bees, quantitative PCR and next generation sequencing are reliable tools, and were already used to prove that the exposure to neonicotinoids alters the expression of acetylcholine receptors, vitellogenin, stress transcripts and transcripts linked to the immune system in honey bees (24). Moreover, global gene expression analysis using next generation sequencing showed negative effects of the insecticides clothianidin, imidacloprid and thiametoxam on metabolism and detoxification (26). In addition, the exposure of honey bees to fungicides such as azoxystrobin and chlorothanolin, and to the insecticide spinosad resulted in altered expression of oxidative phosphorylation transcripts (36, 37). Thus, these substances may also disturb the energy metabolism of honey bees.

**Figure 1:**
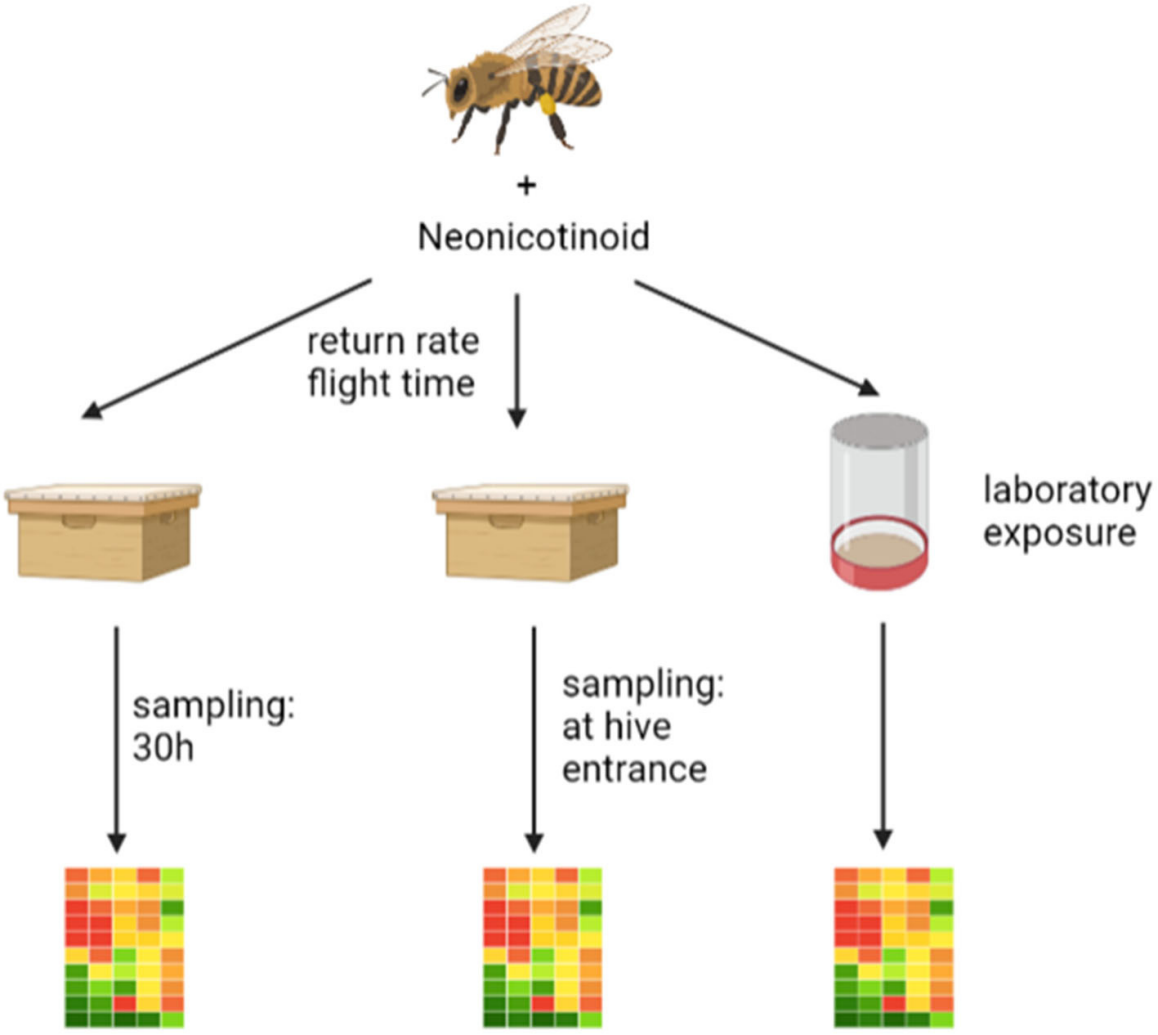
The graphic shows the three experiments performed. On the left is the pilot study (experiment I) where an RFID experiment was performed and the returning foragers were collected after about 30 h and stored frozen until gene expression analysis. In the middle is the second “worst-case” study (experiment II) where an RFID experiment was performed after neonicotinoid exposure and the returning foragers were collected directly from the flight board and stored frozen until gene expression analysis. On the right is the laboratory study (experiment III) where foragers were exposed to neonicotinoids and after an incubation period of about 1 h were frozen until gene expression analysis. The graphic was drawn with the help of biorender (https://Biorender.com).

In our opinion, there are two coherent explanations for the failure of pesticide exposed bees in returning to their hive. Firstly, the orientation could be impaired and thus the path home could no longer be found or detours could be flown (38). Secondly, the energy metabolism in the brain of foragers could be disturbed and thus displaying negative effects on flight behavior. To test the latter hypothesis, we conducted a series of RFID experiments in which we exposed pollen foragers to thiamethoxam- or thiacloprid-laced sugar solutions. Through this experimental design, we monitored both return rates and return durations in combination with gene expression analysis in successfully returned pollen foragers. Thus, we were able to link return times of individual pollen foragers with expression patters, for the first time. In order to understand the underlying molecular mechanisms, we measured transcript levels of genes involved in energy metabolism, endocrine regulation, detoxification and oxidative stress (such as oxidative phosphorylation genes) in pooled and individual bee brain samples. To investigate possible differences between honey bee colonies, the experiments were carried out on foragers originating from different bee colonies. In addition, a standardized laboratory-based exposure was performed independently from an RFID homing flight test to confirm the results obtained under controlled conditions, aiming at developing a functional laboratory-based bioassay. As a further point, in the study presented here, we investigated the effects of group feeding and individual feeding on data variability in gene expression and homing flight ability of treated bees, as the dosing of the treatment might vary due to unequal trophallaxis among caged bees (35, 39). A graphic description of the performed experiments can be seen in Figure 1. In a first pilot study (experiment I), an RFID experiment was conducted with pollen foragers exposed to thiamethoxam. The return rate and the return time were measured. Successfully returning pollen forager bees were sampled 30 h after release and gene expression analyzed in pooled brain samples. At that time, we knew from previous studies that transcripts of endocrine regulation, memory formation, and the detoxification system were altered by neonicotinoids (24, 27). Therefore, we examined the expression of the following transcripts: *hbg-3, ilp-1, vitellogenin, pka, creb, cyp9q1, cyp9q2*, and *cyp9q3*. The goal was to see possible differences in expression patterns between controls and thiamethoxam exposed foragers. Since this was the case, we decided to conduct experiment II and III. Beside the possible negative effects of neonicotinoids on memory formation, we thought that a dysregulation of the transition of nurse bees to foragers might also have negative effects on flight behavior (40). Therefore, we were interested in the expression of transcripts linked to endocrine regulation. In a second “worst-case” study (experiment II), RFID homing flight experiments were conducted with pollen foragers exposed to thiamethoxam and thiacloprid. Again, the return rate and the return time were assessed. Returning foragers were collected directly upon their return, ranked based on their return time and differences in gene expression of fast returning controls and slow returning bees exposed to neonicotinoids were analyzed. Since we had evidence that exposure of bees to pesticides negatively affects energy metabolism (36, 37), we analyzed in experiment II transcripts of oxidative phosphorylation (*cox5a, cox5b, cox6c*, and *cox17*) in addition to transcripts of endocrine regulation (*buffy, hbg-3, ilp-1*, and *vitellogenin*). Since we could only isolate a limited amount of RNA from one brain, we could only analyze a limited number of transcripts. In a third experiment, a standardized, laboratory exposure was performed independently of an RFID test (experiment III) to confirm previous data. Therefore, pollen foragers from three honey bee colonies were exposed to thiamethoxam and thiacloprid and the expression of different transcripts was analyzed immediately after treatment. To verify gene expression data from experiment II, transcripts of oxidative phosphorylation (*cox5a, cox5b, cox6c*, and *cox17)* and endocrine regulation (*hbg-3, ilp-1*, and *vitellogenin)* were again analyzed. In addition, transcripts of the detoxification system (*cyp9q1, cyp9q2*, and *cyp9q3*) and a general stress marker (*catalase*) were analyzed. We were once more able to show effects on transcripts of oxidative phosphorylation genes; however, responses varied between different honey bee colonies. Overall, our results suggest that neonicotinoid exposure of forager bees disturbs their energy metabolism, and this disturbance might cause a reduced return rate or a prolonged flight time in neonicotinoid exposed bees in the RFID homing flight test.

## Materials and Methods

Chemicals: Thiacloprid and thiamethoxam (purities of all >99%) were purchased from Sigma–Aldrich (Buchs, Switzerland). Stock solutions for each compound were prepared in acetone and diluted into 20% sucrose-solution to a final exposure concentration.

### Measuring of Homing Flight Duration and Homing Rate

The homing flight ability test of thiamethoxam or thiacloprid treated honey bees employed here is a ring tested method and described in detail in the OECD guidance document No. 332 (https://www.oecd.org/officialdocuments/publicdisplaydocumentpdf/?cote=ENV-CBC-MONO(2021)7%20&doclanguage=en).

### Preparation of Test Bees

For each run (run: experiment performed with one specific honey bee colony) about 600 returning foragers of one honey bee colony were caught at the hive entrance and marked with a non-toxic pink powder (Pigment Laser Red Fluorescent A3, T series, COLOREY SAS, France). During the collection period of about 2 h, the caught bees were fed *ad libitum* with candy (Apifonda®, Südzucker AG, Germany) and kept in the dark. The powdered bees were subsequently brought to the release location (one kilometer distance to their hive) and released. About 300 returning bees, clearly identifiable by the pink powder on their body, were caught a second time and kept in the dark with access to candy. This pre-selection was necessary to make sure that only forager bees, which were familiar with the environment and oriented well, were used for the test.

### Tagging Individual Bees

The foragers collected during this initial step were then randomly allocated to a treatment group and individually marked with a passive RFID chip of the MAJA 13.56 MHz RFID system (Microsensys GmbH, Germany). The chip was fixed with TempoSIL 2® dental cement (Coltène Holding AG, Switzerland) dorsally on the thorax of each bee.

### Controlled Application of Spiked Feeding Solutions

For the pilot study [experiment I; three independent experiments (runs A, B, and C) performed on different honey bee colonies, each experiment: three group feeding cages per treatment], bees were kept in cages of of ten bees. The bees in each cage were fed with 200 μL of 30% (w/V) sucrose solution (control group), or with 200 μL of 30% (w/V) sucrose solution containing 0.3 ng thiamethoxam/bee, 1 ng thiamethoxam/bee, or 1.5 ng thiamethoxam/bee (treatment groups). Thiamethoxam doses were based on the RFID ring test protocol. It was assumed that an individual bee consumes 20 μL of spiked sucrose solution. However, the total amount of 200 μL sucrose solution was distributed among the ten caged bees *via* trophallaxis (transfer of liquid food from one animal mouth to another as a common behavior in social insects). It was expected that the bees of one group-feeding cage share the offered sucrose solution, however, it was uncertain that the amounts of food was equally distributed among the ten bees in a cage (39). Hence, the dosage of the chemical may vary among the individual bees of a group-feeding cage (e.g., one or some bees receive more of the chemical than the other bees of the same cage). Therefore, a comparison between group feeding and single feeding mode was performed in experiment II.

### Single vs. Group Feeding

For the “worst-case” study (experiment II; three independent experiments (runs A, B, and C) performed on different honey bee colonies, three feeding cages per treatment per experiment, and additionally 30 bees in individual cages per treatment per experiment), two different feeding strategies were applied. Test bees were caged in individual cages receiving 20 μL of 30% (w/V) sucrose solution (control group, abbreviation in graphic: 1er) or 20 μL 30% (w/V) sucrose solution spiked with 1 ng thiamethoxam/bee (treatment group, abbreviation in graphic: 1er) to analyze single bee feeding mode. To analyze group feeding mode, bees were fed in groups of ten bees with 200 μL of 30% (w/V) sucrose solution (control group, abbreviation in graphic: 10er) or with 30% (w/V) sucrose solution containing 1 ng thiamethoxam/bee, 0.25 μg thiacloprid/bee, or 1.25 μg thiacloprid/bee (treatment groups, abbreviation in graphic: 10er). Thiamethoxam dose was based on the RFID Ringtest protocol. Based on the ring-test data 1 ng/bee was considered as sublethal dose with effects on return rate. 1.25 ug thiacloprid was chosen base on literature data (38). 0.25 ug was added as additional lower dose (70 times below oral toxicity; LD_50_ value of thiacloprid: 17.32 μg a.s./bee) to investigate possible sublethal effects on homing behavior.

### Release of Workers and Monitoring of Homing Rate and Flight Duration

After complete consumption of the provided treatment dose (i.e. max. 90 min), the exposed bees were returned to the release site, one kilometer away from their hive, and released a second time. All four entrances of the test bee hive were equipped with RFID readers of the MAJA 13.56 MHz RFID system (Microsensys GmbH, Germany). Returning bees were forced to enter the hive using one of the four entrances and passed the RFID reader upon their return. All bees tagged with an RFID chip that successfully returned to their hive within 24 h after the second release were identified by passing the RFID reader and the exact return time recorded.

### Calculation of Return Times

The return time (homing flight duration) was calculated from the time period between release and triggering of the RFID sensor at the hive entrance. The return time, however, was only quantifiable for bees that were able to return to their hive and passed the RFID readers at the hive entrance. The homing flight test was repeated twice for each experiment. For experiment I and II, three runs each were performed on individual days with six different bee colonies. The weather conditions for each test run were optimal for the homing flight test (mean daily temperature for start of the bees at the release site and the following 48 h between 24 and 28°C and no precipitation). For the analysis of return rates and return times, IBM® SPSS® Statistics version 26 software (IBM Corporation, USA) was used to evaluate statistical significant differences between treatment groups (resulting *p* < 0.05 were considered as statistically significant) for each experiment individually. Differences between return rates of treatment groups were tested with pairwise, two-sided, Welch-*t*-tests. The differences in the duration of time needed to return to the hive were evaluated by applying non-parametric Kruskal-Wallis rank tests. We used the Bonferroni method to correction for multiple comparisons. For the comparison of group feeding and single feeding mode employed in experiment II, a generalized linear mixed model followed by Bonferroni correction for multiple comparisons was used to test for statistical differences between treatment groups.

### Laboratory Study Without RFID Flight Phase

For the laboratory based exposure study (experiment III, done at three different days using bees of three different colonies: run A, B, and C), 400 foragers were collected when returning to their hive, distributed in four boxes and colored with 30 mg of pink powder (pigments fluorescents- serie T, COLOREY SAS) per box. Subsequently, colored bees were fed *ad libitum* with bee candy/sugar paste (Apifonda®, Südzucker AG, Germany) for ~20 min and were transported to the release site one km away from the hive. Returning powdered foragers were collected and kept in three individual cages and fed with bee candy/sugar paste (Apifonda®, Südzucker AG, Germany) for 1 h. Foragers were handled as in the RFID experiments, whereby in contrast only dental glue was placed on the bee thorax without RFID tag. During labeling, no food was supplied. Bees were individually fed with 20 μL of test solutions each (*n* = 45 individuals for each group). Bees were kept in darkness in an incubator, at 25°C and 60% relative humidity for 1 h. To simulate the transport to the release site (see protocol RFID experiment), bees were transported for some minutes in a car followed by sacrifice and storage at −20°C until gene expression analysis. The experiment was conducted on three individual days on 20 foragers per treatment originating from three different honey bee colonies.

### Gene Expression Analyzes

For experiment I, successfully returned honey bees were collected 30 h after release and subjected to gene expression analysis, whereas in the second “worst-case” study (experiment II), bees were sampled directly after their arrival at the hive entrance. The number of bees used and the replicates that were analyzed are listed in Supplementary Table 1.

RNA isolation, reverse transcription and qPCR: The brain of frozen bees was removed in total by opening the cranium using scalpel and forceps. Total RNA was isolated (experiment I and III: two brains pooled for one RNA sample, experiment II: one brain used for one RNA sample) using RNeasy®Mini Kit (Qiagen, Basel, Switzerland) according to the manufacturer's instructions. Thousand nanogram RNA was reverse transcribed as described before (24). Furthermore, qPCR based on SYBR green fluorescence (SYBR green PCR master mix; Roche, Rotkreuz, Switzerland) was performed as described in 24. Primer sequences used in this study are given in Table 1. For all performed gene expression analyzes *ribosomal protein S5* (*rpS5*) was used as housekeeping gene for normalization. This selection was based on the stable transcription of *rpS5* across seasons in a previous study (47). Alterations of mRNA abundance in neonicotinoid exposed brain samples were always compared to solvent control (acetone) samples to determine the effects of neonicotinoids.

**Table 1:** Primer sequences of transcripts analyzed using qPCR.

**Function**	**Primer**	**Direction**	**Sequence**	**Analyzed in Experiment**	**References**
Housekeeping gene	*Ribosomal protein s5 (rps5)*	Forward Reverse	AATTATTTGGTCGCTGGAATTG TAACGTCCAGCAGAATGTGGTA	I, II and III	(41)
Oxidative phosphorylation	*Cytochrome c oxidase subunit 5A (cox5a)*	Forward Reverse	TCGCATGATGGACCACAAGA AGGTACAAGATCCAGCCGC	II and III	(36)
	*Cytochrome c oxidase subunit 5B (cox5b)*	Forward Reverse	TGGATGTGGTTACATGATGGC AAAGTGGTGCAACTTGAGTAAG	II and III	(36)
	*Cytochrome c oxidase subunit 6C (cox6c)*	Forward Reverse	TCGCTTACAGAACACATCTACA ACGAAGCTGAGGCTTTGGTAA	II and III	(36)
	*Cytochrome c oxidase copper chaperone (cox17)*	Forward Reverse	AACCTTGTTGTGCTTGT ATGTGCTTCTATTAAATCCC	II and III	(36)
Endocrine regulation	*Buffy*	Forward Reverse	CATGGCACTTCTCATCCTTTT GAGAACGGTTTCAGCATCAAT	II	(42)
	*αGlucosidase (hbg-3)*	Forward Reverse	TACCTGGCTTCGTGTCAAC ATCTTCGGTTTCCCTAGAGAATG	I, II and III	(42)
	*Insulin-like peptide 1 (ilp-1)*	Forward Reverse	GCTCAGGCCTGTGCTCGAAAAGT CGTTGTATCCACGACCCTTGC	II and III	(43)
	*Vitellogenin*	Forward Reverse	GCAGAATACATGGACGGTGT GAACAGTCTTCGGAAGCTTG	I, II and III	(44)
Memory formation	*Cyclic AMP-responsive element-binding protein 1 (creb)*	Forward Reverse	CGATGCAGCACCAGCAATAG AGTCTCAACCACCTGAAGCG	I	(24)
	*cAMP-dependent protein kinase catalytic subunit α (pka)*	Forward Reverse	AAGACTATTGAAGTCGGTGACA CCTATCAAGGCCCCACCAAA	I	(24)
Detoxification	*Cytochrome p450 9q1 (cyp9q1)*	Forward Reverse	TCGAGAAGTTTTTCCACCG CTCTTTCCTCCTCGATTG	I and III	(45)
	*Cytochrome p450 9q2 (cyp9q2)*	Forward Reverse	GATTATCGCCTATTATTACTG GTTCTCCTTCCCTCTGAT	I and III	(45)
	*Cytochrome p450 9q3 (cyp9q3)*	Forward Reverse	GTTCCGGGAAAATGAATC GGTCAAAATGGTGGTGAC	I and III	(45)
Stress	*Catalase (cat)*	Forward Reverse	GGCGGGTGAATTAAGTGCTA TTGCGTTGTGTTGGAGTCAT	III	(46)
	*Heat shock protein 90 (hsp90)*			I	

Data processing and statistical analysis: To check the normal distribution of the raw data, a Shapiro-Wilk-Test was performed. Differences between treatments were assessed by one-way ANOVA (including F test to analyze the homogeneity of variance) followed by a Bonferroni‘s multiple comparison correction to compare treatment means with respective controls. Results of transcripts are given as means ± standard error of means. Differences were considered statistically significant and marked with one asterisk at 0.05 > *p* > 0.01, or two asterisks at 0.01 > *p* > 0.001 or three asterisks at 0.001 > *p* > 0.0001. Correlation between homing flight duration and gene expression was analyzed using linear regression analysis with r^2^: goodness of fit and analysis of significance (significantly non-zero) with *p* ≤ 0.05.

## Results

The study presented here consists of three experiments that build on each other. The exact sequence of the three experiments is shown in Figure 1.

### Pilot Study: Experiment I

**Figure 2:**
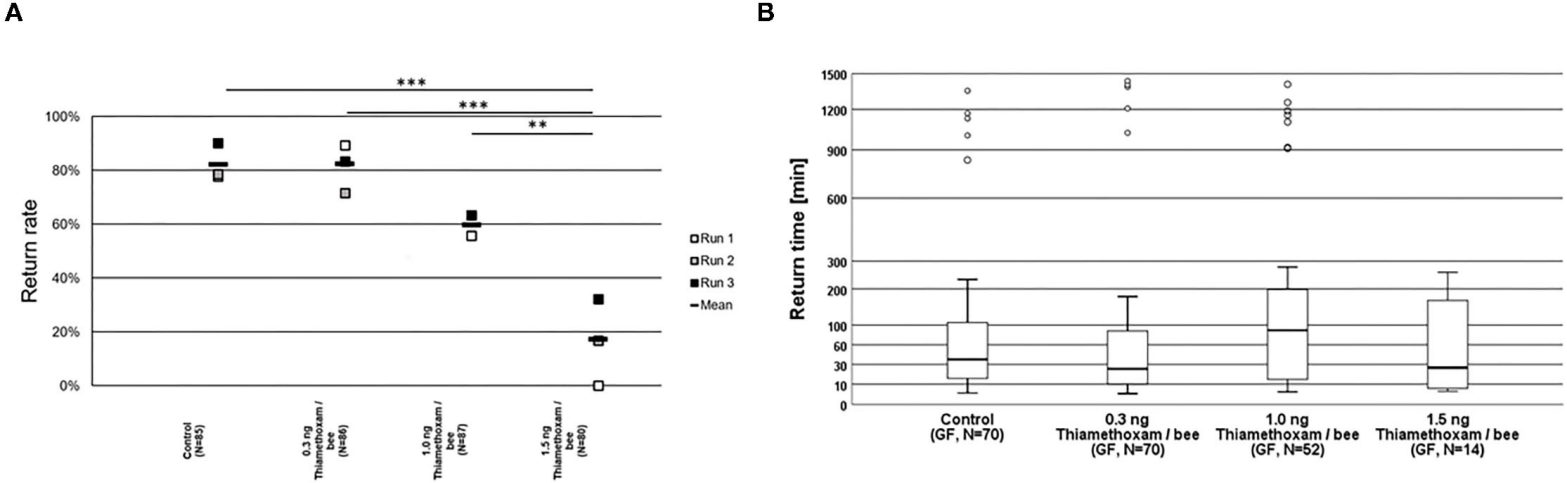
Return rates and return times of bees in the RFID test exposed to thiamethoxam at doses of 0.3, 1.0, or 1.5 ng/bee applying group feeding mode. **(A)** Each square represents the percentage of returning bees. Asterisks on top mark statistical significant differences between treatments (two-sided Welch-*t*-test with Bonferroni correction, *p < 0.05, **p < 0.01, ***p < 0.001). **(B)** Return times: The y-axis is depicted in exponential scale and circles depict outliers as return times varied substantially. No statistical significant differences between treatment groups in the return times were detected (Kruskal-Wallis test with Bonferroni correction for multiple pairwise comparisons, *p < 0.05, **p < 0.01, ***p < 0.001).

The homing flight return rates of honey bees treated with the highest tested dose of 1.5 ng thiamethoxam/bee, was considerably lower and significantly different to those return rates observed in non-treated control bees, or in the groups treated with the lower thiamethoxam doses at 0.3 and 1.0 ng thiamethoxam /bee (Figure 2A). Return times were varying strongly within treatment groups, the first bees arrived at the hive entrance about 4 min after their release one km away from the hive, whereas the last successfully returning bees passed the RFID reader at the hive entrance 1,434 min after release. Untreated control bees needed 36.6 min in median to re-entering their hive entrance, bees treated with 0.3, 1.0, or 1.5 ng thiamethoxam showed median return time of 24.6, 88.6, and 26.1 min, respectively. No statistical significant differences between treatment groups in the return times were detected (Kruskal-Wallis test with Bonferroni correction for multiple pairwise comparisons, *p* > 0.05; Figure 2B).

As barely any foragers exposed to 1.5 ng/bee thiamethoxam were able to successfully return to their hive, gene expression was only analyzed in foragers exposed to 0.1 and 1 ng/bee thiamethoxam. The expression of transcripts linked to endocrine regulation (*hbg-3, hsp-90, ilp-1*, and *vitellogenin*), to memory formation (*creb* and *pka*) and detoxification (*cyp9q1, cyp9q2*, and *cyp9q3*) was analyzed in returned foragers, collected 30 h after their release. RFID experiments were performed three times (run A, B, and C) applying group feeding approach. No significant changes were detected in the expression of *hbg-3* and *hsp90*. *Ilp-1* showed significant upregulation in run B at 0.1 and 1 ng/bee thiamethoxam and in run C at 0.1 ng/bee thiamethoxam and in the combination of all three runs at 0.1 ng/bee thiamethoxam. *Vitellogenin* showed significant up-regulation in run B at 0.1 ng/bee thiamethoxam (Supplementary Figure 1). A significant induction of *creb* expression was detected in bees of run B treated with 0.1 ng/bee thiamethoxam and also with the combination of all three runs at 0.1 ng/bee thiamethoxam. *Pka* showed significant downregulation at 0.1 ng/bee thiamethoxam in run C (Supplementary Figure 2). *Cyp9q1* was significantly induced at 0.1 ng/bee thiamethoxam in run B and in the combination of all runs. In contrast, *cyp9q2* was significantly inhibited at 0.1 ng/bee thiamethoxam in run C. The expression of *cyp9q3* did not show any significant changes (Supplementary Figure 3).

### Second “Worst-Case” Study: Experiment II

**Figure 3:**
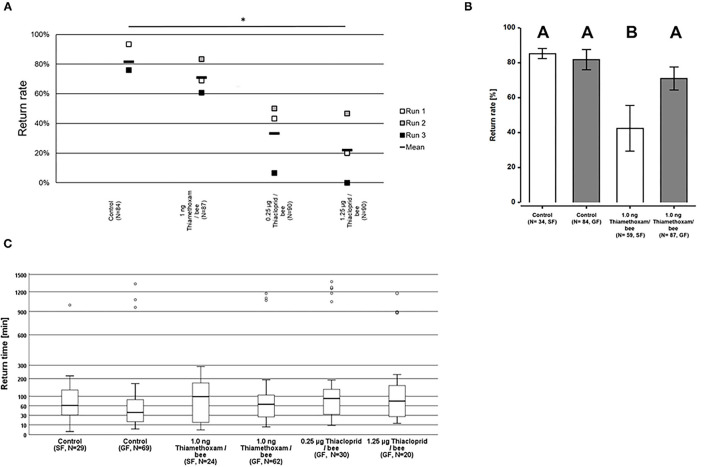
Return rates and return times of bees in the RFID test exposed to 1.0 ng thiamethoxam /bee or 0.25 or 1.25 μg thiacloprid/bee in single bee feeding or group feeding mode. **(A)** Return rates of bees applying group feeding mode. Each square represents the percentage of returning bees per individual run. Asterisks on top mark statistical significant differences between treatments (two-sided Welch-*t*-test with Bonferroni correction, *p < 0.05, **p < 0.01, ***p < 0.001). **(B)** Return rates of bees in the RFID test exposed to 1.0 ng thiamethoxam /bee in single feeding (SF, white bars) or group feeding (GF, gray bars) mode. Each bars represents the mean percentage (Mean ± SE) of returning bees. Significant differences in return rates are indicated by different letters on top of bars (Generalized linear mixed model with Bonferroni correction for multiple comparisons, *p < 0.05, **p < 0.01, ***p < 0.001). **(C)** Return times of honey bees after treatment with different doses of thiamethoxam in single feeding (SF) of group feeding (GF) mode or thiacloprid (only group feeding mode) in the RFID homing flight test. The y-axis is scaled exponentially as return times varied substantially, and circles above boxplots depict outliers. No statistical significant differences between treatment groups in the return times were detected (Kruskal-Wallis test with Bonferroni correction for multiple pairwise comparisons, *p < 0.05, **p < 0.01, ***p < 0.001).

The homing flight success rates were not significantly altered in bees exposed to either 1.0 ng thiamethoxam/bee, or 0.25 μg thiacloprid/bee in the group feeding mode, compared to unexposed control bees (Two-sided Welch-*t*-test with Bonferroni correction, *p* > 0.05, Figure 3A). This result corresponds to the results of the pilot study where we also found no differences in the homing flight success of bees treated with 1.0 ng thiamethoxam/bee compared to the untreated controls. However, the treatment with the higher dose of 1.25 μg thiacloprid/bee had a significant effect on the homing ability of bees, as the return rates of bee after this treatment was significantly lower to all other tested treatments (Two-sided Welch-*t*-test with Bonferroni correction, *p* < 0.05, Figure 3A). Interestingly, the feeding mode also had an effect on the return rates of the tested bees. Whereas, non-treated control bees had the same homing success irrespective of the feeding mode, bees treated with 1.0 ng thiamethoxam/bee in the single feeding mode failed more often to return to their hive compared to bees fed with the same dose of thiamethoxam in the group feeding mode or untreated control bees (Two-sided Welch-*t*-test with Bonferroni correction, *p* < 0.05, Figure 3B). This supports our hypothesis that single dosing is more accurate than group feeding *via* trophallaxis, in consequence this strongly suggests that single feeding is advantageous over group feeding if sublethal doses of chemical substances are tested on honey bees.

Return times varied strongly within treatment groups, the first bees arrived at the hive entrance about 4 min after their release 1 km away from the hive, whereas the last successfully returning bees passed the RFID reader at the hive entrance only 1,430 min after release. The median times for the return to the hives varied for the treatment groups between 37.8 and 89.7 min for returning to the hive, however, no statistical significant differences between treatment groups in the return times were detected (Kruskal-Wallis test with Bonferroni correction for multiple pairwise comparisons, *p* > 0.05; Figure 3C).

**Figure 4:**
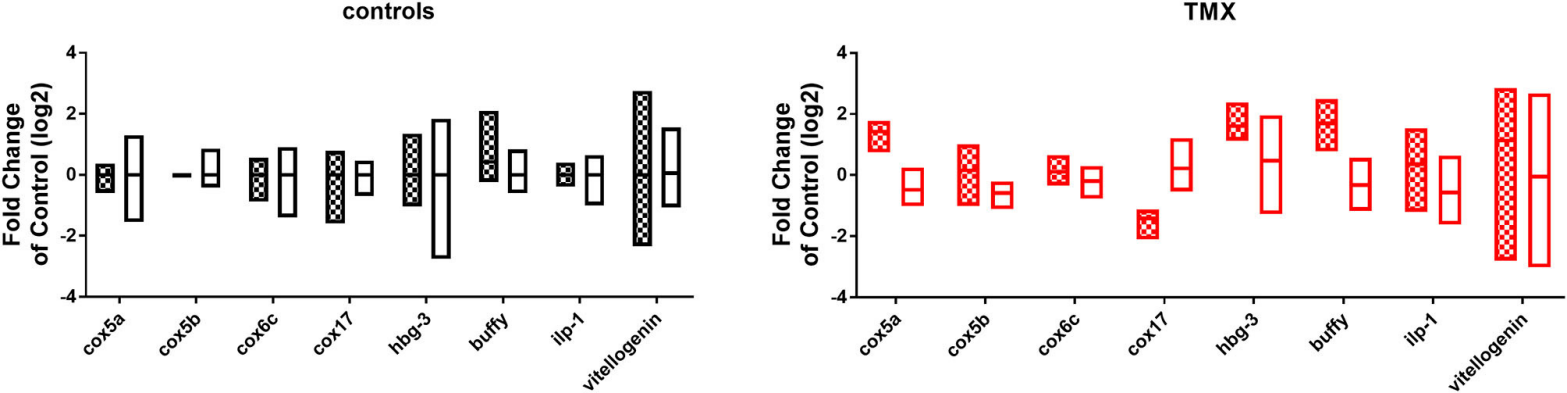
Comparison of gene expression data of all analyzed transcripts of controls (left graph, bars: black and white) and thiamethoxam (TMX) exposed foragers (right graph, bars: red and white) of single feeding (bars with squares) and group feeding (bars without pattern).

**Figure 5:**
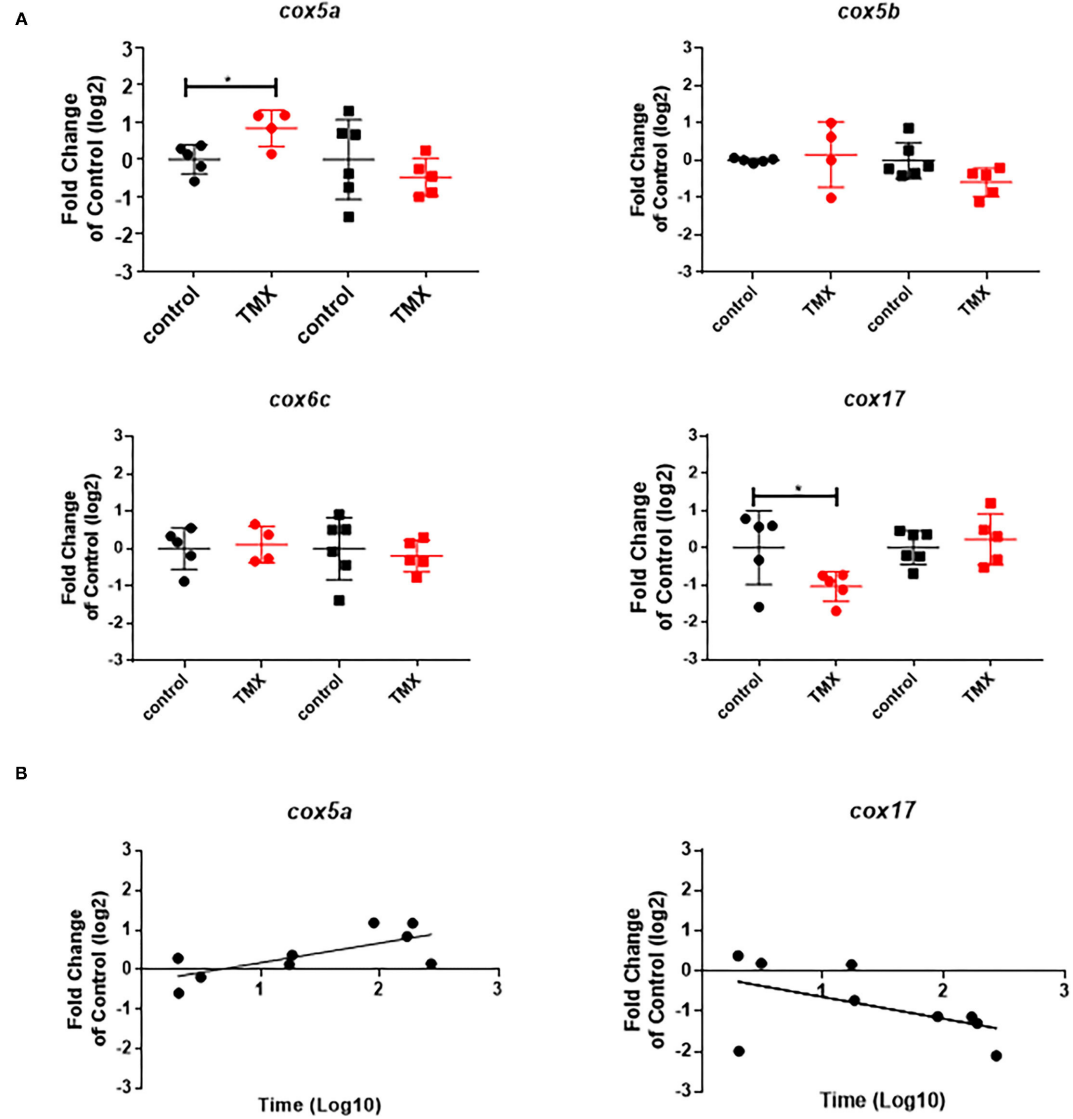
**(A)** Abundance of transcripts of *cox5a, cox5b, cox6c*, and *cox17* in the brain of foragers of the RFID experiment applying two different feeding approaches (single bee feeding: dots, group feeding: squares) and exposed to sugar syrup (black) and 1 ng/bee thiamethoxam (red). Significant differences with *p*-value of ≤0.05 (*cox5a*: 0.0244; *cox17*: 0.0468) are marked with asterisks. **(B)** Correlation between expression of *cox5a* (r^2^: 0.51; F: 7.354; *p*: 0.0301) and *cox17* (r^2^: 0.26; F: 2.489; *p*: 0.1587) and homing flight duration.

To link changes in gene expression to homing flight duration, bees were ranked for their time needed to return to their hive and fast returning controls (homing flight duration <25 min) and slow returning exposed foragers (homing flight duration >85 min) were selected for gene expression analysis to create a “worst-case” scenario. Unfortunately, there were few thiacloprid exposed foragers among the collected bees, therefore gene expression analysis was performed only with thiamethoxam exposed bees. The number of analyzed bees per treatment is summarized in Supplementary Table 1. Flight times of selected foragers are displayed in Table 2, Supplementary Figure 4. Mean flight time of selected controls was 8.57 (single bee feeding) and 15.24 min (group feeding) and of thiamethoxam exposed foragers 180.10 min (single bee feeding) and 136.30 min (group feeding). To analyze the possible effects of feeding method on data scattering, gene expression between single bee feeding and group feeding of all treatments was compared. The samples of the group feeding (controls and thiamethoxam exposed bees) showed greater variation than samples of the individual feeding. Only in the case of *vitellogenin* no difference was found. Here, the data for both, group and individual feeding, were extensively scattered (Figure 4) and no alterations in gene expression were detected applying group feeding approach (Figure 5A, Supplementary Figure 5). Exposure to thiamethoxam led to a significant up-regulation of *cox5a* and down-regulation of *cox17* using single bee feeding. The other analyzed transcripts did not show any significant changes when applying single bee feeding (Figure 5A, Supplementary Figure 5). To investigate the correlation between flight time and gene expression, a linear regression was performed for *cox5a* and *cox17*. Since there is an outlier for *cox17*, no correlation between flight time and *cox17* expression could be detected. The expression of *cox5a* increases significantly with the duration of the flight time (Figure 5B).

**Table 2:** Flight times of selected foragers of “worst-case” study (experiment II).

**Run**	**Sample number**	**Treatment**	**Flight time (minutes)**
3	181	Control, single bee feeding	2.01
	182		18.34
	186		3.12
	194		17.36
	213		2.04
	160	Thiamethoxam, single bee feeding	271.41
	171		189.51
	180		170.14
	214		89.14
	157	Control, group feeding	22.48
	178		8.08
	179		6.55
	204		17.55
	206		13.27
	209		23.55
	175	Thiamethoxam, group feeding	187.36
	189		144.48
	190		104.26
	197		105.02
	205		140.38

### Laboratory Study: Experiment III

**Figure 6:**
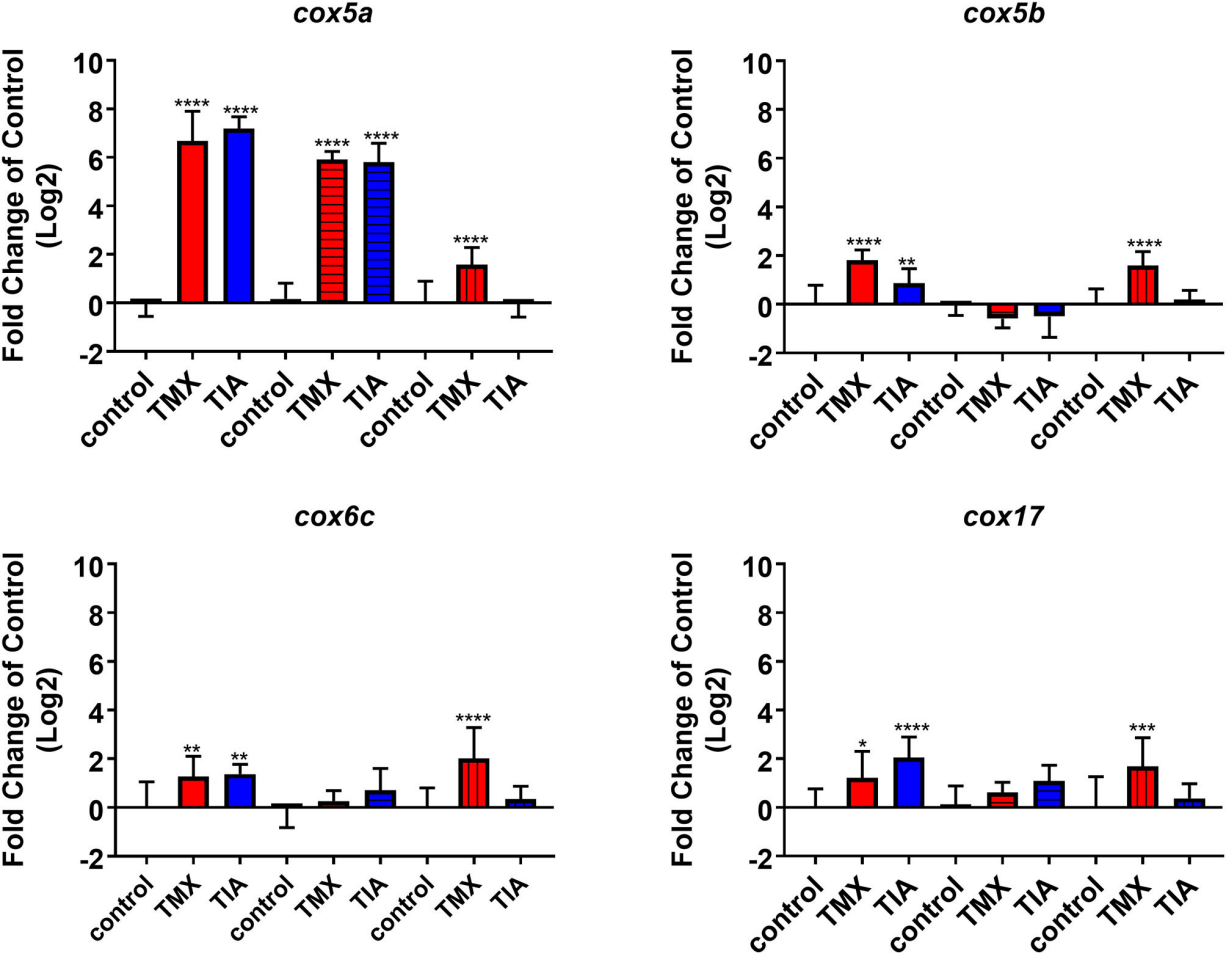
Abundance of transcripts of *cox5a, cox5b, cox6c*, and *cox17* in the brain of foragers (*n* = 10) of three different hives (run A: without pattern, run B: cross-striped and run C: lengthwise striped) exposed to 1 ng/bee thiamethoxam (red) and 8 ng/bee thiacloprid (blue) applying single feeding approach. Significant differences between treatments and controls with *p*-value of ≤0.05 (*cox5a*: all *p* < 0.0001; *cox5b*: < 0.0001, 0.0076, and < 0.0001; *cox6c*: 0.0668, 0.002, and <0.0001; *cox17*: 0.0221, <0.0001 and 0.007) are marked with asterisks.

**Figure 7:**
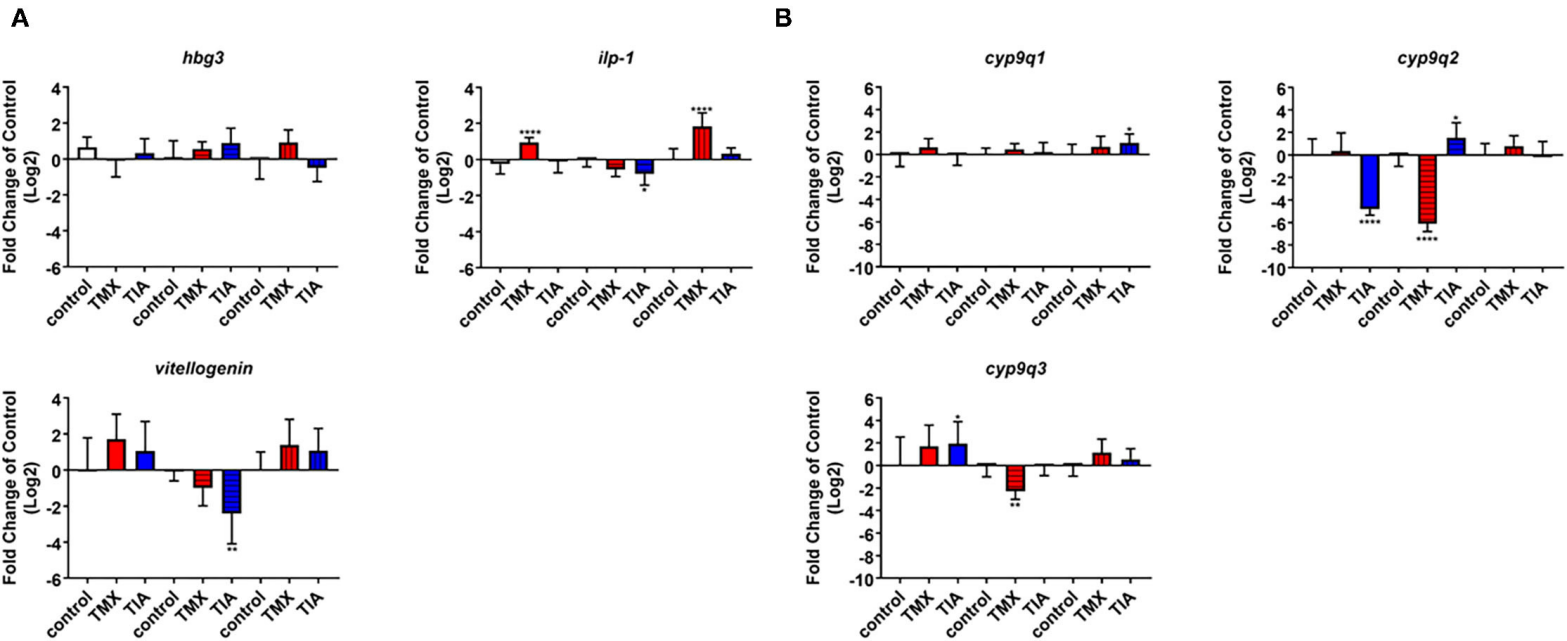
Abundance of transcripts of *hbg3, ilp-1* and *vitellogenin* **(A)** and *cyp9q1, cyp9q2*, and *cyp9q3* **(B)** in the brain of foragers (*n* = 10) of three different hives (run A: without pattern, run B: cross-striped and run C: lengthwise striped) exposed to 1 ng/bee thiamethoxam (red) and 8 ng/bee thiacloprid (blue) applying single feeding approach. Significant differences between treatments and controls with *p*-value of ≤0.05 (*ilp-1*: < 0.0001, 0.0266 and <0.0001; *cyp9q1*: 0.0413; *cyp9q2*: <0.0001, <0.0001, and 0.0447; *cyp9q3*: 0.0247 and 0.0075) are marked with asterisks.

**Figure 8:**
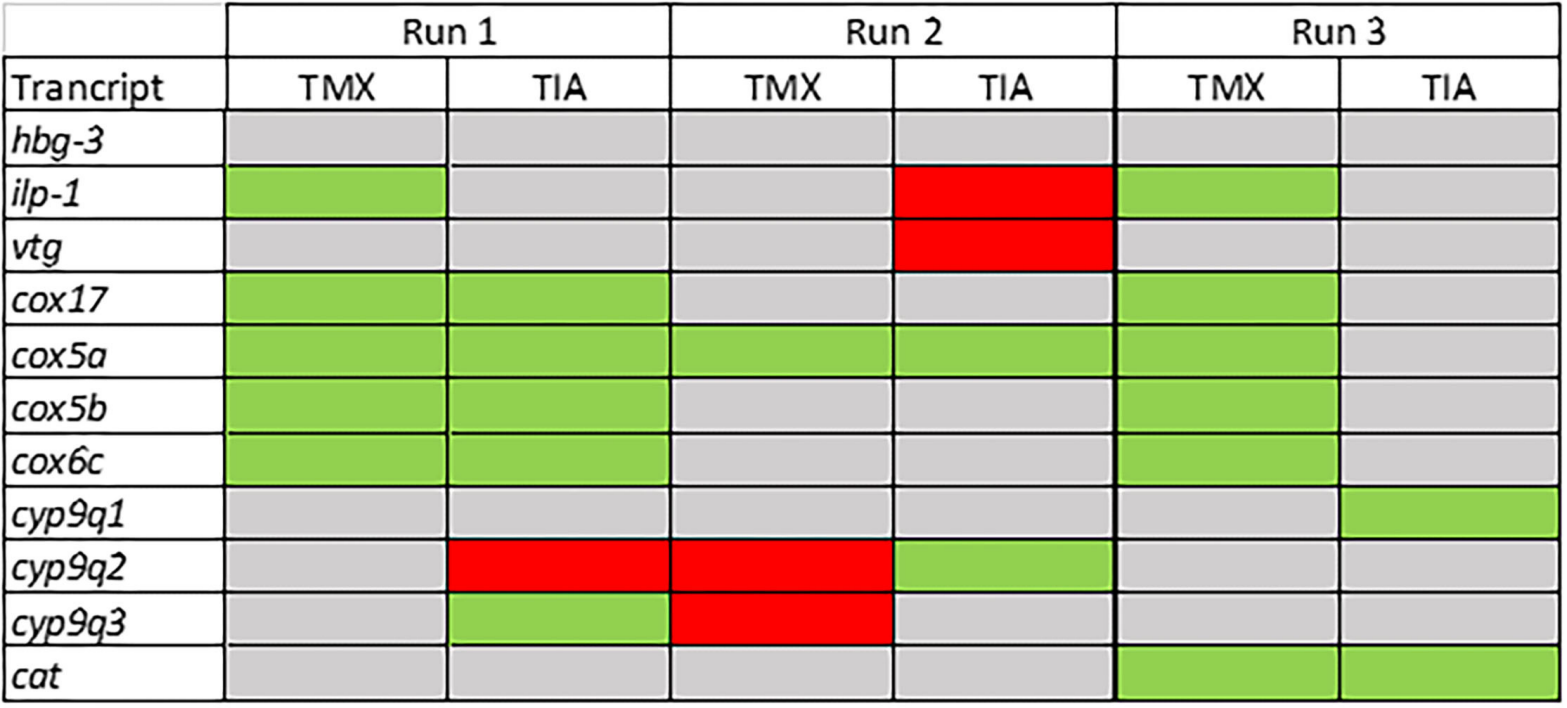
Summary of the transcriptional data of the forager bees from three different colonies (run A, B, and C; experiment III). Significant down-regulations are shown in red, up-regulations in green and no changes in gray.

To investigate the effects of thiamethoxam and thiacloprid on the expression of genes involved in energy metabolism and endocrine regulation in more detail, foragers from three different colonies (run A, B, and C) were exposed to thiamethoxam and thiacloprid in the laboratory applying single bee feeding. After 1 h exposure and a short car trip, bees were frozen until further analysis. The expression of transcripts linked to energy metabolism, endocrine regulation, detoxification and oxidative stress was analyzed. The expression of *cox5a* was significantly up-regulated in foragers exposed to thiamethoxam of run A, B, and C and in foragers exposed to thiacloprid of run A and B. *Cox 5b* was significantly up-regulated in foragers exposed to thiamethoxam of run A and C and in foragers exposed to thiacloprid of run A. Thiamethoxam induced *cox6c* significantly in foragers of run A and C, thiacloprid in foragers of run A. *Cox17* was significantly induced in foragers of run A and C after thiamethoxam exposure and in foragers of run A after thiacloprid exposure (Figure 6). No alteration in the expression of *hbg3* was found after exposure to thiamethoxam or thiacloprid. *Ilp-1* was significantly up-regulated in foragers of run A and C after exposure to thiamethoxam and down-regulated in foragers of run B after exposure to thiacloprid. In addition, thiacloprid inhibited significantly the expression of *vitellogenin* in foragers of run B (Figure 7A). The expression of transcripts linked to detoxification was also significantly altered. *Cyp9q1* was up-regulated in foragers of run C after thiacloprid exposure. *Cyp9q2* was down-regulated in foragers of run A after thiacloprid exposure and in foragers of run B after thiamethoxam exposure. In addition, it was up-regulated in foragers of run B after thiacloprid exposure. *Cyp9q3* was up-regulated in foragers of run A after thiacloprid exposure and down-regulated in foragers of run B after thiamethoxam exposure (Figure 7B). The expression of the stress marker *catalase* was significantly induced in foragers of run C after exposure to thiamethoxam and thiacloprid (Supplementary Figure 6). To get an overview of all transcriptional changes, a heat map was made. It is evident that foragers from different honey bee colonies also react differently to neonicotinoid treatment, with foragers from run A and C showing similar expression patterns. In contrast to this, foragers of run B show a different expression pattern. Strong expression changes after both thiamethoxam and thiacloprid exposure can be observed in transcripts linked to oxidative phosphorylation. Effects on transcripts of endocrine regulation and detoxification are less strong (Figure 8).

## Discussion

In the study presented here, we aimed at finding answers to the following questions: Is there a measurable difference in gene expression of forager bees exposed to neonicotinoids and non-treated control bees, which were previously tested in RFID homing flight experiments? Is there a correlation between homing flight duration and gene expression in the brain of foragers after exposure to neonicotinoids? Can we identify possible biomarker transcripts that indicate altered flight behavior after exposure to neonicotinoids? Are there differences in the response to neonicotinoid exposure between different bee colonies? Does the type of feeding, individual or group feeding, influence the data quality/scatter? To answer the first question, we analyzed gene expression in the brains of returned unexposed and exposed foragers in the performed RFID experiment. We detected differences in the expression of various transcripts, such as *vitellogenin, ilp-1, creb, cyp9q1*, and *cyp9q2* (Supplementary Figures 1–3), although most significant effects showed no dose dependence. In addition, the individual RFID-runs showed different expression patterns. An altered expression of *vitellogenin* and *creb* in the brain of foragers after neonicotinoid exposure has already been shown in another study (24). Effects of neonicotinoids on endocrine regulation, and thus on the expression of endocrine-relevant transcripts such as *ilp-1* after neonicotinoid exposure, have also been shown (27). In these studies, most detected effects did not show dose dependence, as observed in this study. With these initial experiments, we were able to confirm published data on effects of neonicotinoids on the gene expression of bees (24, 27) and demonstrate that foragers from the RFID experiment are useful study objects to perform gene expression analyzes. The prolongation of the flight time of foragers after neonicotinoid exposure may result, among other things, from the fact that the orientation of the bees and the memory formation are disturbed and/or that the energy metabolism is disturbed and therefore too little energy is available for flying. In the literature, both theories are confirmed: On one hand, several studies show negative effects of neonicotinoids on the orientation of honey bees supporting the first theory. The exposure of foragers to thiamethoxam resulted in impaired orientation and longer flight duration (48). Exposure of foragers to imidacloprid, clothianidin, and thiacloprid resulted in reduced return rates and fewer direct returns from the food source to the hive (38). Exposure of foragers to clothianidin, imidacloprid and thiamethoxam led to altered expression of *creb* and *pka*, two transcripts linked to memory formation (24). Furthermore, some studies have shown that exposure of bees to pesticides impair the energy metabolism and that neonicotinoids and other pesticides disturbed mitochondrial function (22, 36, 37, 49). Exposure of bees to nicotine leads to an upregulation of proteins of the energy metabolism [oxidative phosphorylation, proteins of complexes I, III and IV; (50)]. This upregulation serves to meet the increased energy demand through detoxification of nicotine by cytochrome P450 enzymes (50). Exposure of honey bees to pyraclostrobin has negative effects on mitochondrial activity, leading to inhibition of oxidative phosphorylation and altered mitochondrial membrane potential. This is particularly critical for the energy supply of foragers (49). Exposure of honey bees to different fungicides such as azoxystrobin, chlorothanolin and folpet leads to altered expression of transcripts such as *cox5a, cox5b, cox6c, cox17*, and *ndufb7*. These transcripts encode proteins of complex I and IV of oxidative phosphorylation (36). Spinosad, an insecticide used in organic farming, also leads to altered expression of oxidative phosphorylation transcripts in the brain of foragers (37). In order to investigate the relationship between prolonged flight time and impaired energy metabolism after neonicotinoid exposure of sampled bees, the expression of oxidative phosphorylation transcripts in the brain of bees used in the RFID homing flight test was investigated. Gene expression analysis was performed on the brain of individual bees to establish a direct correlation between expression pattern and flight time. In contrast to the pilot study, foragers were frozen directly after returning to the bee hive to analyze gene expression pattern at the time of return to the bee hive and not as in the pilot study only several hours later. To establish a “worst-case” scenario, the fast-returning controls and the very slow-returning thiamethoxam-exposed foragers were selected for gene expression analysis (Supplementary Figure 4). In slowly returning thiamethoxam-exposed foragers, the expression of *cox5a* was significantly increased and the expression of *cox17* was significantly decreased (Figure 5A). There was a weak, but statistically significant correlation between up-regulation of *cox5a* expression and prolongation of homing flight duration (Figure 5B). The change of expression of transcripts linked to oxidative phosphorylation after exposure to pesticides is in accordance to already published studies (36, 37). These data support the hypothesis that exposure of foragers to neonicotinoids has negative effects on energy metabolism and therefore might cause prolonged return time in these bees. The expression of *cox5a* may be used in future studies as biomarker of disturbed homing flight activity. However, it must be mentioned that in this study, fast-returning unexposed control bees were compared with slow-returning exposed bees. This approach assumes that all bees have the same energy metabolism before exposure and therefore the same flight ability. This assumption is certainly oversimplified and in follow-up experiments, gene expression in slow-flying non-exposed bees should therefore also be analyzed. In addition, the comparison between individual feeding and group feeding showed that data generated from group feeding fluctuated more than data generated from individual feeding (Figure 4). Due to this, no significant effects were detected in foragers of the group feeding approach (Figure 5). When several bees are fed in a cage (group feeding), single bees take up the food and then distributes it to the other bees in the cage. This phenomenon is known as trophallaxis. Therefore, the food containing the tested substance offered to the bees is not evenly distributed among all bees within the cage. Hence, when honey bees are exposed to pesticides *via* sucrose solution during group feeding, different exposure concentrations may occur in individual bees (39). This finding is important in relation to existing OECD guidelines. For the determination of acute oral toxicity, the OECD 213 guideline suggests feeding in groups of 10 bees. Based on the findings of the present study, this should be reconsidered with respect to data quality and reproducibility. To support the hypothesis of disturbed energy metabolism after neonicotinoid exposure, to confirm the obtained data and to analyze differences between different honey bee colonies the third experiment was conducted. Foragers from three different colonies were exposed to thiamethoxam and thiacloprid and then the expression of different transcripts was analyzed. When comparing experiment II to III, it must be noted that in experiment II, the bees flew in addition to neonicotinoid exposure (two possible stressors) while the bees of experiment III were exposed only to neonicotinoids (one stressor). Foragers from colony 1 and 3 showed a significant upregulation of *cox5a, cox5b, cox6c*, and *cox17* after thiamethoxam exposure. Thiacloprid induced *cox5a, cox5b, cox6c*, and *cox17* only in foragers from colony 1. Foragers from colony 2 showed a completely different response to exposure to thiamethoxam and thiacloprid (Figure 8). As the mitochondrial activity changes during the transition from nurse bees to foragers and the capacity of oxidative phosphorylation in the head of foragers is lower compared to nurse bees (51), effects of neonicotinoids on the expression of transcripts of the oxidative phosphorylation may have tremendous effects on energy metabolism in foragers. This experiment demonstrated that exposure to neonicotinoids can lead to altered expression of oxidative phosphorylation transcripts. In particular, the expression of *cox5a* was altered. However, it also clearly shows that transcriptional changes vary greatly when comparing different honey bee colonies. Based on the data shown here, *cox5a* may be a potential biomarker for altered homing flight duration. However, further studies are needed to confirm this.

## Conclusion

In the study presented here, first, it was shown that exposure of foragers to neonicotinoids has a negative impact on return rates. Secondly, it could be shown that neonicotinoid exposure has an influence or sublethal effect on gene expression and that a different expression pattern were identified especially in relation to transcripts of energy metabolism between fast and slow returning foragers. There was a correlation between flight duration and *cox5a* expression. The altered expression of *cox5a* could be partially confirmed in the laboratory study. Based on the data presented here, a laboratory test can be developed for the future in which sublethal effects (for example flight behavior) can be predicted based on gene expression patterns. Furthermore, for the risk assessment of plant protection products for bees, such a laboratory test could be established as a first test approach triggering higher tier studies, e.g., a homing test (OECD guidance document No. 332) in a risk assessment scheme to evaluate sublethal effect on honey bees. Before such a laboratory test can be developed, more transcription data must be available. Therefore, studies applying next generation sequencing are needed to identify the exact gene expression patterns, as it is difficult to make assumptions based on expressional changes of only a few transcripts. Third, it has been shown that feeding mode has a or significant effect on the variability of the data. This should be considered in the development of future guidelines. Another important point that should be considered in future guidelines is the fact that it has been clearly shown that different colonies respond differently to the same exposure. A larger study with more colonies should be performed to confirm this hypothesis.

## Data Availability Statement

The original contributions presented in the study are included in the article/Supplementary Material, further inquiries can be directed to the corresponding.

## Author Contributions

Homing flight experiments were done by DG, LJ, and ME. Gene expression analysis was done by VC. J-DC supervised the work and was involved in the experimental design. DG, LJ, and VC contributed all in the same way to write the manuscript. All authors contributed to the article and approved the submitted version.

## Conflict of Interest

The authors declare that the research was conducted in the absence of any commercial or financial relationships that could be construed as a potential conflict of interest.

## Publisher's Note

All claims expressed in this article are solely those of the authors and do not necessarily represent those of their affiliated organizations, or those of the publisher, the editors and the reviewers. Any product that may be evaluated in this article, or claim that may be made by its manufacturer, is not guaranteed or endorsed by the publisher.
