# Supplementary material for: Correlation Between Increased Homing Flight Duration and Altered Gene Expression in the Brain of Honey Bee Foragers After Acute Oral Exposure to Thiacloprid and Thiamethoxam
Source: Front Insect Sci. 2021 Dec 10;1:765570. doi: 10.3389/finsc.2021.765570 (PMC10926505; doi:10.3389/finsc.2021.765570)
Supplement: Supplementary file 1 [file Data_Sheet_1.PDF]

Supplementary materials

Correlation between impaired homing flight activity and altered gene expression in the brain of honey bee foragers after acute oral exposure to thiacloprid and thiamethoxam

Verena Christen[1], Daniela Gossar[2], Jean-Daniel Charrière[2], Michael Eyer[3] and Lukas Jeker[2]

1 University of Applied Sciences and Arts Northwestern Switzerland, School of Life Sciences, Hofackerstrasse 30, CH-4132, Muttenz, Switzerland.

2 Agroscope, Swiss Bee Research Center, Schwarzenburgstrasse 161, CH-3003 Bern, Switzerland

3 Laboratory of Soil Biodiversity, University of Neuchâtel, 2000 Neuchâtel, Switzerland

Table S1: Numbers of used bees for gene expression analysis of the three experiments (I, II and III)

Figure S1: Abundance of transcripts of *hbg-3*, *ilp-1*, *hsp90* and *vitellogenin*

Figure S2: Abundance of transcripts of *creb* and *pka*

Figure S3: Abundance of transcripts of *cyp9q1*, *cyp9q2* and *cyp9q3*

Figure S4: Flight times of selected controls and thiamethoxam exposed foragers

Figure S5: Abundance of transcripts of *buffy*, *hbg3*, *ilp-1* and *vitellogenin*

Figure S6: Abundance of *catalase*

Table S1: Numbers of used bees for gene expression analysis of the three experiments (I, II and III)

Experiment 1	Run A	Run B	Run C
control	14 > pooled to 7 RNA samples	14 > pooled to 7 RNA samples	14 > pooled to 7 RNA samples
0.1 ng/bee TMX	14 > pooled to 7 RNA samples	14 > pooled to 7 RNA samples	14 > pooled to 7 RNA samples
1 ng/bee TMX	10 > pooled to 5 RNA samples	8 > pooled to 4 RNA samples	14 > pooled to 7 RNA samples
Experiment 2			Run C
Control-single bee feeding			5 > 5 RNA samples of one brain each
Control-group feeding			5 > 5 RNA samples of one brain each
TMX- single bee feeding			4 > 4 RNA samples of one brain each

TMX- group feeding			5 > 5 RNA samples of one brain
Experiment III	Run A	Run B	Run C
Control	20 > pooled to 10 RNA samples	20 > pooled to 10 RNA samples	20 > pooled to 10 RNA samples
1 ng/bee TMX	20 > pooled to 10 RNA samples	20 > pooled to 10 RNA samples	20 > pooled to 10 RNA samples
8 ng/bee TIA	20 > pooled to 10 RNA samples	20 > pooled to 10 RNA samples	20 > pooled to 10 RNA samples

Fig. S1:

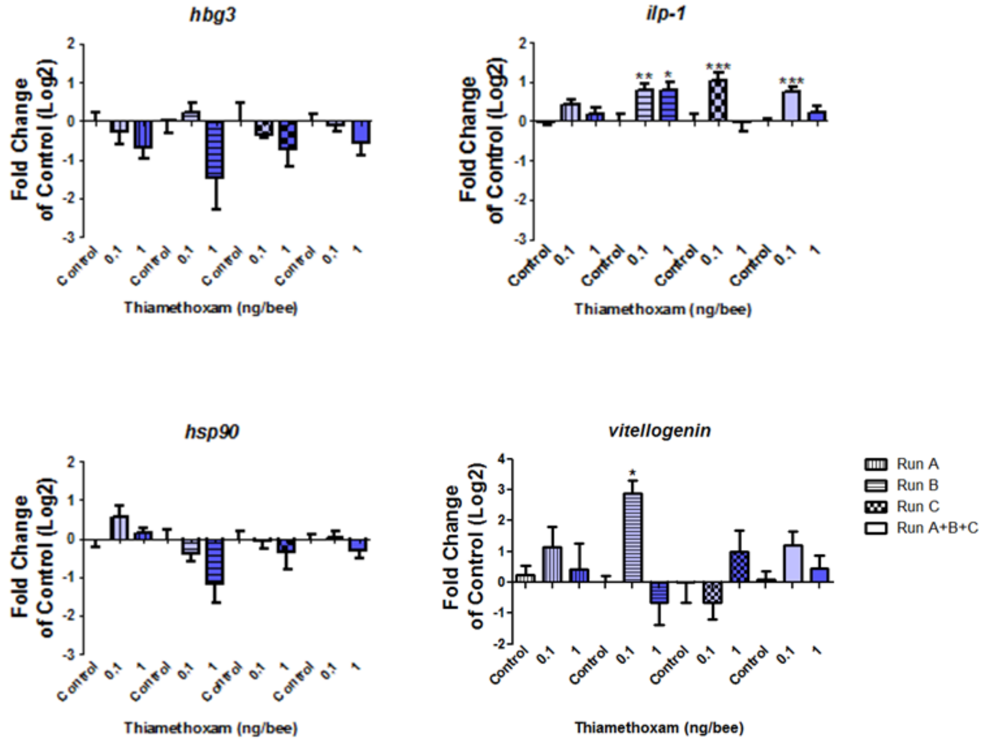

Figure S1: Abundance of transcripts of *hbg-3*, *ilp-1*, *hsp90* and *vitellogenin* in the brain of foragers of the three different RFID experiments (run A: lengthwise striped, run B: cross-striped, run C: squared and all runs combined: without pattern). Shown are the results of: controls and 0.1 ng/bee thiamethoxam n= 7; 1 ng/bee thiamethoxam run A: n=5, run B: n=4 and run C: n=7. Significant differences with p-value of ≤ 0.05 are marked with asterisks.

Fig. S2:

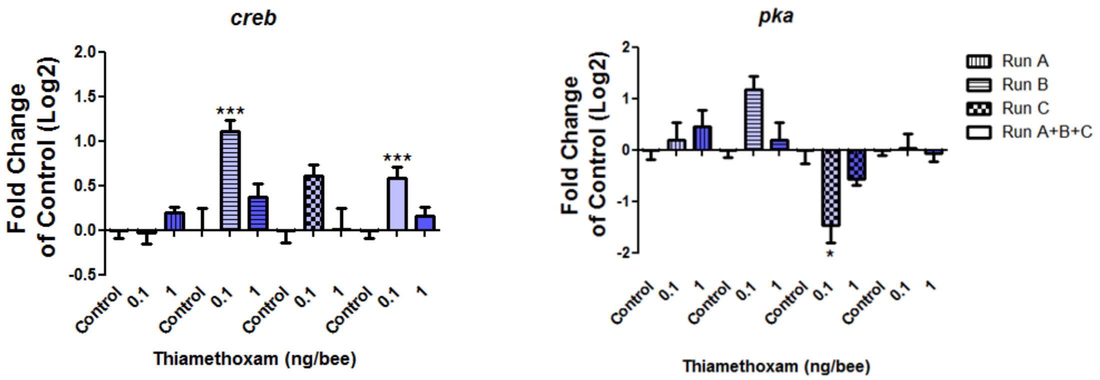

Figure S2: Abundance of transcripts of *creb* and *pka* in the brain of foragers of the three different RFID experiments (run A: lengthwise striped, run B: cross-striped, run C: squared and all runs combined: without pattern). Shown are the results of: controls and 0.1 ng/bee thiamethoxam n= 7; 1 ng/bee thiamethoxam run A: n=5, run B: n=4 and run C: n=7. Significant differences with p-value of ≤ 0.05 are marked with asterisks.

Fig. S3:

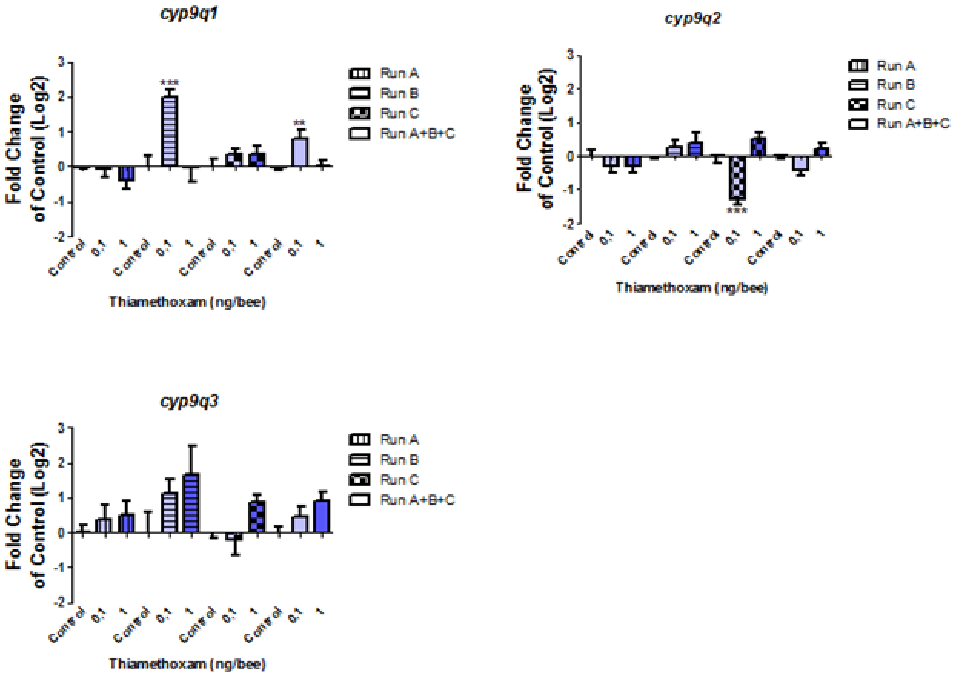

Figure S3: Abundance of transcripts of *cyp9q1*, *cyp9q2* and *cyp9q3* in the brain of foragers of the three different RFID experiments (run A: lengthwise striped, run B: cross-striped, run C: squared and all runs combined: without pattern). Shown are the results of: controls and 0.1 ng/bee thiamethoxam n= 7; 1 ng/bee thiamethoxam run A: n=5, run B: n=4 and run C: n=7. Significant differences with p-value of ≤ 0.05 are marked with asterisks.

Fig. S4:

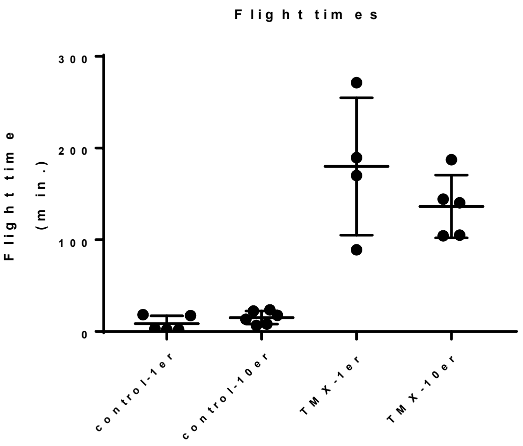

Figure S4: Flight times of selected controls and 1 ng/bee exposed thiamethoxam foragers (1er: single feeding and 10er: group feeding).

Fig. S5:

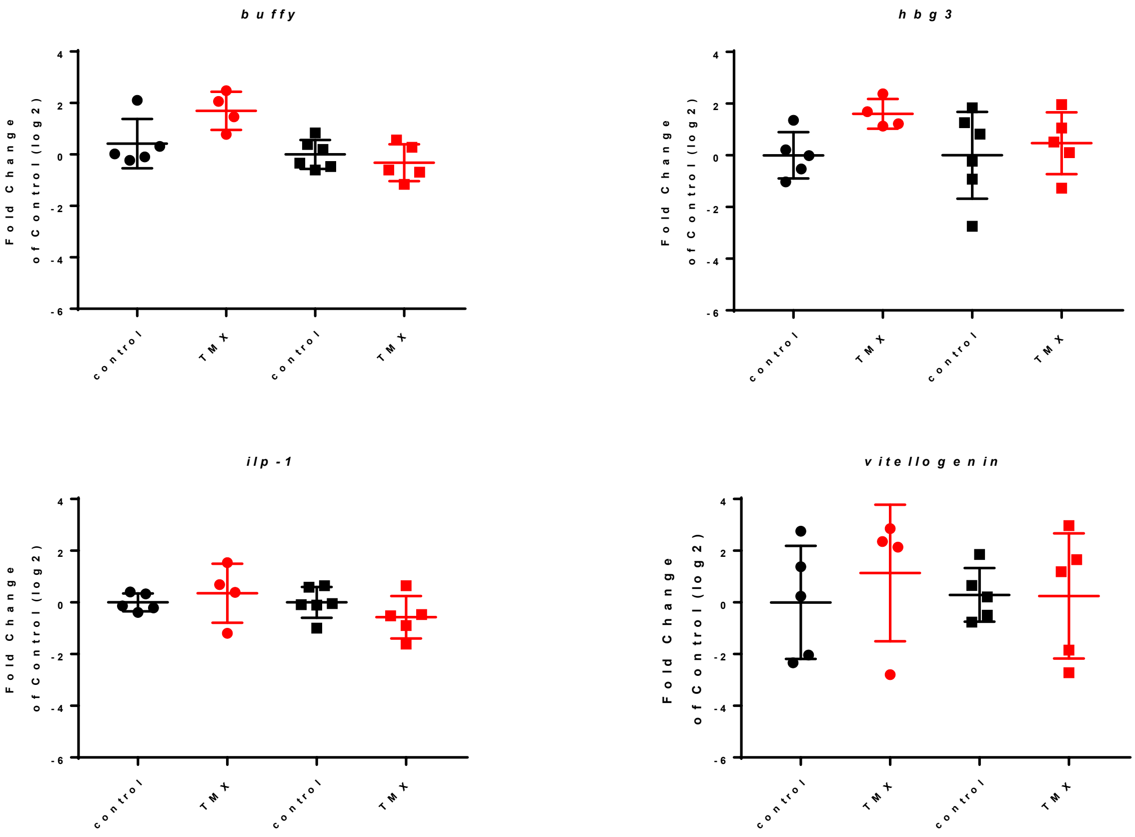

Figure S5: Abundance of transcripts of *buffy*, *hbg3*, *ilp-1* and *vitellogenin* in the brain of foragers of the RFID experiment applying two different feeding approaches (single bee feeding: dots, group feeding:

squares) and exposed to sugar syrup (black) and 1 ng/bee thiamethoxam (red). Significant differences with p-value of ≤ 0.05 are marked with asterisks.

Fig. S6:

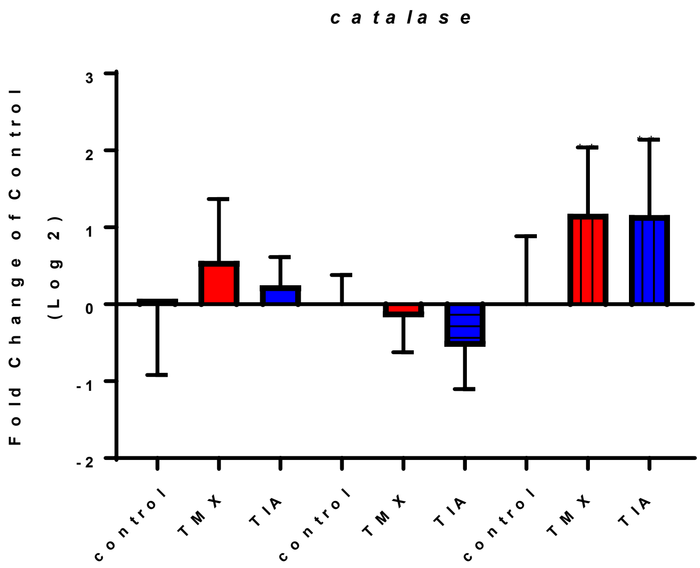

Figure S6: Abundance of *catalase* in the brain of foragers (n=10) of three different hives (run 1: without pattern, run 2: cross-striped and run 3: lengthwise striped) exposed to 1 ng/bee thiamethoxam (red) and 8 ng/bee thiacloprid (blue) applying single feeding approach. Significant differences between treatments and controls with p-value of ≤ 0.05 are marked with asterisks.
